# The Communication Challenges and Strength of Nurses’ Intensive Corona Care during the Two First Pandemic Waves: A Qualitative Descriptive Phenomenology Study

**DOI:** 10.3390/healthcare10050837

**Published:** 2022-05-02

**Authors:** Gizell Green, Cochava Sharon, Yulia Gendler

**Affiliations:** Nursing Department, Ariel University, Ariel 40700, Israel; cochavas@ariel.ac.il (C.S.); yuliage@ariel.ac.il (Y.G.)

**Keywords:** intensive care, nurses, communication, COVID-19, patients

## Abstract

Intensive care nurses working with patients with severe COVID-19 illness are at the center and frontline of the dynamic pandemic, which poses communication challenges and demands of unusual strength on their part. The study aim was to capture the lived experience of intensive care COVID nurses’ communication challenges and strengths as they cared for COVID-19 intensive care patients during the two first pandemic waves. The study used qualitative descriptive-phenomenology research designs. Twenty-two nurses were selected using snowball sampling, and online interviews were conducted with them. Data were recorded and transcribed, then reflexively double-coded for increased rigor. Four major themes emerged from the data. The first two expressed the communication challenges and difficulties communicating with patients due to the extreme protection needed and/or their medical condition. However, the other two themes expressed the nurses’ strengths—sharing feelings with other caregivers and family. Accordingly, we recommend using simple language and ensuring patient comprehension, as well as creating an optimistic environment for fostering caregiver bonding.

## 1. Introduction

Since December 2019, an increasing number of cases of a novel coronavirus (COVID-19/SARS-Cov-2) have been identified. These cases were first reported in Wuhan, a large city in central China [1], which later rapidly spread both domestically and internationally, including to Israel [1,2]. On 30 January 2020, the WHO held an emergency meeting and declared the global COVID-19 outbreak a public health emergency of international concern and, on 11 March, recognized it as a pandemic [3].

In the first wave in Israel, there were 500–700 new proven cases per day. Furthermore, Israel had a death rate of fewer than 300 individuals, representing 33 deaths per million, and a fatality rate of 1.67%, much lower than in the majority of European states [4]. However, in June 2020, the extent of new proven cases of COVID-19 began to grow, to 4000–6000 new cases per day in the first half of September. The number of deaths reached approximately 1200 or 130 deaths per million. At this point, with a total of almost 180,000 proven cases, 50,000 of which were active, Israel became the most infected country in the world. This happened in the second wave [4].

Facing this critical situation, health care workers on the front line who are directly involved in the diagnosis, treatment, and care of patients with COVID-19 are at risk of developing psychological distress and other mental health symptoms [5]. The ever-increasing number of confirmed and suspected cases, overwhelming workload, depletion of personal protection equipment, widespread media coverage, lack of specific drugs, and feelings of being inadequately supported may all contribute to the mental burden of these health care workers [6].

Previous studies have reported adverse psychological reactions to the SARS or Ebola outbreaks among health care workers. Research shows that these health care workers operated under high-stress levels [7,8], reported reluctance to work or the contemplation of resignation, and experienced symptoms of anxiety and depression, which could have long-term psychological implications [7,9]. Great distress was also associated with the need for quarantine and interpersonal isolation [10].

Similar concerns have been raised about the mental health, psychological adjustment, and recovery of health care workers caring for patients with COVID-19 [5,11]. Throughout the pandemic period, repeated reports were received regarding a significant association between the COVID-19 outbreak and adverse mental health issues, such as stress, burnout, depression, and anxiety among healthcare workers [12,13].

Studies have shown that the severity and fatality of COVID-19 have caused and intensified anxiety and fear among nurses, affecting their health, well-being, and work effectiveness [12]. In addition, frontline nurses, particularly those working directly with COVID-19 patients, often witness patients suffering and dying, impacting their emotional health and causing compassion fatigue and post-traumatic stress manifestations [14,15].

Intensive Care Corona Unit (ICCU) nurses were especially susceptible to these phenomena, being at the center and frontline of defusing the crisis of the COVID-19 pandemic. Evidence suggests that over 80% of ICCU nurses were at high risk of burnout, compassion fatigue, loss of resilience, and emotional exhaustion due to the pandemic [16]. In addition, during the pandemic period, many nurses became infected with COVID-19 and even died as a result. An overwhelming work overload required staff mobility among departments, and regular ward nurses were sometimes forced to work in the ICCU. Working in an unfamiliar environment further increased the sense of frustration and burnout among nurses, who were afraid to work with unfamiliar equipment and make a mistake that would cost human lives [16,17].

The sources of stress varied throughout the pandemic. The first major sources of stress were a lack of personal protective equipment and fear of becoming infected or dealing with the new disease [18,19,20]. Subsequently, new challenges arose, such as communication difficulties with patients. Wearing facemasks led to fewer facial cues and compromised the nurses’ ability to express and recognize emotional cues [21]. Nurses in the ICCU found it difficult to communicate with patients who were ventilated or connected to extracorporeal membrane oxygenation (ECMO) [22]. However, communication can also be a source of strength for nurses.

Previous studies have revealed the need for high-level communication support for nurses in terms of providing up-to-date information regarding the pandemic and providing clear and concise work protocols [20]. Chen et al. (2021) described the psychological support offered to frontline nurses through target groups and conversations with mental health professionals [23,24]. Although frontline nurses experienced a variety of mental health challenges, particularly burnout and fear, they refrained from discussing this in support groups and with management [25]. They usually drew the needed emotional support from colleagues, friends, and family members [26].

Hence, a profound understanding of the situation the nurses face daily while working with COVID-19 patients and supporting the nursing workforce during the pandemic is of paramount importance for the current epidemic management and especially as a prerequisite for unexpected crisis times that may occur in the future in Israel, which can serve an example for other countries. Accordingly, our study aims to describe nurses’ experience with the phenomenon of communication challenges and strengths as they cared for COVID-19 intensive care patients during the two first pandemic waves, considered crisis times in Israel.

## 2. Materials and Methods

### 2.1. Research Design

A qualitative descriptive-phenomenology research design was used [27] to explore the experiences of communication challenges and strengths of nurses caring for COVID-19 intensive care patients during the two first pandemic waves. Descriptive-phenomenology research design illustrates persons’ everyday life experiences, as well as the values of these experiences, as explained by those who live them [28]. This method was chosen in this research to reveal the experiences of nurses caring for patients diagnosed with COVID-19 in Israel regarding communication features.

### 2.2. Participants and Procedure

The participants of the present study were intensive care nurses working in Israeli hospitals that were voluntarily recruited by the researcher through email and text messaging. The inclusion criteria were nurses with a B.A. degree working in general intensive care who had been moved to work in a COVID intensive care unit. Exclusion criteria included novice nurses and nursing students with no experience in an intensive care unit.

The participants were 22 male and female nurses providing intensive care nursing to COVID-19 patients in the isolation units. The mean age was 38.20 (SD = 5.20) years. The average time working at the ICCU was 7 months (SD = 3). Their background details are presented in Table 1.

Table 1 shows that most of the participants were female. In addition, most of them were married. Half of them had an undergraduate degree, and the other half had graduate degrees. Furthermore, most of them worked as regular nurses. 

During the pandemic outbreak, the nurses’ working environment was rife with stressful conditions. Hence, in order to reduce the strain and enable them to undergo the interviews in a comfortable and neutral place for them and reduce COVID-19 transmission risk for both parties [29], online interviews were done via Zoom™ [30] and were audio- and video-recorded, with notes written by the interviewer. The online interviews were scheduled according to the nurses’ times and technologies accessibilities in an attempt to listen to their views and perspectives regarding their experience of providing extensive care to COVID-19 patients. For this purpose, 22 nurses were selected using snowball sampling. The first ‘round’ of recruitment (two nurses) was done through a forum of intensive care nurses via social media. They were the anchor, and, from there, the recruitment continued through snowball sampling.

### 2.3. Research Tools

The research tool used in the study was the semi-structured interview, seeking to examine in depth the communication aspects and experience of nurses caring for COVID-19 intensive care patients during the two first pandemic waves. The interviewer was independent and without preliminary acquaintance with research participants to evade any biases that may evolve from the dual roles of researchers and participants. Interviews were conducted via Zoom™ at the participants’ convenience and were recorded (both audio and video). An interview guide was constructed before the interview to guarantee the collection of relevant information (see Table 2). Throughout the interview, the researcher encouraged the participants to share their experiences and discuss specific points contributing according to their views.

### 2.4. Data Collection and Analysis

In this study, all interviews were done according to the explanation of the research aim—to describe nurses’ experience of the phenomenon regarding communication challenges and strengths as they cared for COVID-19 intensive care patients during the two first pandemic waves. Consent was gained to perform the online interview and to publish the data with no identifiable details. The first section of the interview was closed socio-demographic questions, and the second section, open-ended questions relating to the research aim, with extra questions evolving from the interaction between interviewer and nurse [31]. Questions in the topics included experience and challenges in communication regarding caring for patients during the two first pandemic waves, concerns, and expectations of the pandemic’s future process.

Thematic methodology analysis was used [32] by three independent researchers who examined the transcriptions (I.H., R.T., and O.G.). We used the seven-step framework for critical analysis [33]. In the first stage, the transcription, a good quality audio recording was conducted, as well as an accurate (word-for-word) transcription of the interview. In the second stage, the researchers became familiarized with the interview. In the third stage, coding, after familiarization, the researchers carefully read the transcript and applied a code that described what they interpreted as important. In the fourth stage, developing a working analytical framework, after coding the first few transcripts, all the involved researchers met and compared the labels and received agreement on a set of codes that were grouped into categories by using a tree diagram, eventually referring to a working analytical framework. In the fifth stage, the analytical framework was applied by using the existing categories and codes. At the sixth stage, data were charted into the framework matrix. In the seventh stage, the data were interpreted.

Researchers found possible codes on an independent basis, compared thoughts, and discussed these codes. Once the framework was agreed upon, this was applied to all transcripts. The inductive procedure enabled the detection of the leading thoughts. Finally, subthemes were collected into a higher level of conceptual themes, which were confirmed and refined as the analysis continued [34]. The analysis continued until no new themes arose [35]. The findings of the participants were described qualitatively and quantitatively (frequency and percentage). The findings were summed up alongside existing literature, thus evaluating whether the findings were compatible or incompatible with existing literature data.

### 2.5. Trustworthiness

To ensure the trustworthiness of this study, another experienced researcher with similar competencies evaluated the text to confirm that there was no bias during the analysis and theme development process. Methodological issues were also discussed to ensure trustworthiness.

### 2.6. Ethical Considerations

Approval for the study was obtained from the IRB (Institutional Review Board) of the university (no. 20210104). Previous to data collection, the researchers guaranteed that all participants had read and signed the online written informed consent and understood the aim of the study. The researchers ensured data confidentiality and guaranteed that the information would be published anonymously. The participants were provided with the opportunity to ask questions and seek clarifications before and after the interviews. The online interviews were quiet and held in convenient home environments. For a properly executed conversation in each interview, the interviewer and participant were alone.

## 3. Findings

**Figure 1:**
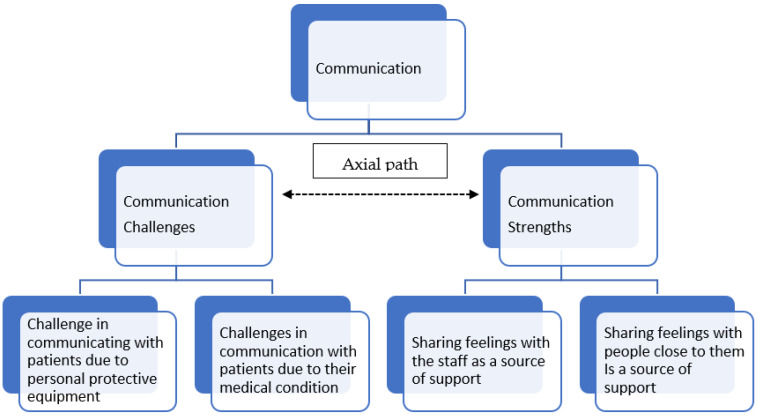
Hierarchy of themes.

The results describe nurses’ experience of the phenomenon regarding communication challenges and strengths as they cared for COVID-19 intensive care patients during the two first pandemic waves (Figure 1).

The ‘meta-theme’ communication that emerged from the interviews’ analysis is multidimensional and complex. On one hand, nurses can see it as a challenge; however, on the other hand, they can see it as a source of strength. ICCU nurses described both pros and cons regarding communication while they cared for COVID-19 intensive care patients during a pandemic, as the ‘path analysis’ in the quotes below demonstrated.

“At the beginning of the pandemic, I felt a great struggle with the communication with patients, however as time went on I also started to see the positive side regarding my ability to communicate with the staff....suddenly they started to understand me almost without any words, it improves my work with them. We became a winning team.”

(ICCU nurse, 29 years old, 8 months experience.)

Another nurse said:

“You can see the communication competence in two ways. One view is that it is very difficult to communicate with patients with respiratory aids. However, another view is that it makes us, as a team, more bonded and more qualified.”

(ICCU nurse, 35 years old, 5 months experience.)

Moreover, the data analysis process yielded four sub-themes. Two of these described the possible challenges in communicating with patients, such as the difficulties presented by the extensive protective gear worn and the patients’ medical condition. The other two themes described the communication-related strengths acting as support mechanisms—sharing with nurses and sharing with the family. Below are quotes from the interviews illustrating the different themes.

### 3.1. Communication Challenges

This topic came up often in the interviews. The participants expressed their frustration with the fact that they could not communicate with the patients like they wanted to or believed they should in a patient-caregiver relationship. These challenges were divided into two. The first was a technical problem due to the protective gear used by the nurses, which made it difficult to communicate reissues such as facial expressions, body language, lip-reading, and hearing, which the nurses tried to overcome by using large hand gestures or writing on a whiteboard. The second challenge was the difficulty in communicating with patients due to their (often serious) medical condition. Most patients were ventilated or almost non-responsive and using ventilation aids, all conditions standing in the way of patient-caregiver communication.

### 3.2. Challenge in Communicating with Patients Due to Personal Protective Equipment (PPE)

The caregivers who used head-to-toe protective gear felt they had difficulty expressing themselves both verbally and physically and making themselves understood to the patients; thus, they made special efforts to bridge this gap. A nurse says:

“I found it difficult to breathe and hear. Work was very difficult because I could not see well because of the mask, there was steam. When I spoke to the patients and tried to communicate with them, they couldn’t really see my facial expression, I felt I had to shout for them to hear and understand me.”

(ICCU nurse, 26 years old, 9 months experience.)

The caregivers emphasized that any type of communication, even the most basic, such as reading body language, was challenging, and often faulty. Another ICCU nurse claimed:

“I begin my shift and care for people, but they cannot see my face, my smile, they cannot see my mouth speaking, this is frustrating. I’m talking about basic communication, the most basic impressions, even that was faulty.” (ICCU nurse, 29 years old, 8 months experience.) The communication-related effort was so great that it made the caregivers uncomfortable.

Another nurse said:

“Communication was very difficult, very challenging. I had a shield and an N-95 mask, so it was almost impossible to speak. My mouth was dry, and I longed for air… The patients found it very difficult to hear us. Most of the time I wasn’t sure whether or not they had understood what I said.”

(ICCU nurse, 37 years old, 5 months experience.)

The nursing staff often used other gestures to aid communication, such as body language and writing on a whiteboard. One of the participants described the situation thus:

“The mask made it very difficult for me to speak. I am used to verbal communication together with facial expressions, presenting myself in a manner best for the patient, and suddenly they couldn’t see my face and it was difficult for them to hear me. I used a lot of hand gestures, and sometimes wrote what I wanted to say on a whiteboard. There is no doubt that there was much difficulty in interpersonal communication.”

(ICCU nurse, 35 years old, 10 months experience.)

### 3.3. Challenges in Communication with Patients Due to Their Medical Condition

Additional communication challenges existed due to the patients’ medical conditions. Most patients used ventilation aids and, thus, could not be understood, making communication extremely challenging.

A nurse worried about patient welfare said:

“When I was in the COVID-19 ward, patients were anesthetized and ventilated, so there was no communication between us, and I couldn’t understand what they were feeling. There were very few patients whose medical condition allowed them to communicate.”

(ICCU nurse, 35 years old, 8 months experience.)

Some of the nurses spoke and communicated, even though they were not sure the patients could understand them, for the sake of the smallest doubt. One nurse said:

“Most patients were anesthetized and ventilated, so it was extremely difficult and challenging to communicate. With the few patients who were not ventilated, I identified myself by the name, I presented myself. I explained that they could not see me. But with the ventilated patients, I don’t know… but I said that I was there, and I could hear, and they could speak to me.”

(ICCU nurse, 37 years old, 5 months experience.)

Being unable to understand the patients due to their medical condition frustrated the nurses as they wanted to provide the best care possible, and communication is part of such care. One nurse claimed:

“You take care of them and speak to them but you don’t know what they can hear or understand if anything… Most of them are ventilated and connected to ventilation aids such as a ventilation machine… People don’t see faces, don’t see a smile, you don’t know whether or not they are in pain, whether or not the treatment is helping, they can’t say… this is extremely frustrating.”

(ICCU nurse, 26 years old, 9 months experience.)

“Of course, there was no real communication, most patients had already been anesthetized and ventilated.”

(ICCU nurse, 37 years old, 5 months experience.)

Another nurse quotes:

“It is known that compassion and empathy contribute to a patient’s recovery; however, I found it difficult to communicate compassionately and empathetically and not knowing whether the patient received it.”

(ICCU nurse, 27 years old, 5 months experience.)

### 3.4. Communication Strengths

Communication has many strengths, and the nursing staff used these as a support system. Through communication, the staff shared their feelings with other nurses and family, thus allowing them to share the emotional burden. The COVID-19 pandemic introduced many challenges, and many of the nurses decided to unburden themselves by sharing their feelings with the medical staff or with their families, particularly their spouses.

### 3.5. Sharing Feelings with the Staff as a Source of Support

The staff felt that the people who can understand the best are the ones who had undergone similar experiences, i.e., the hospital staff. They felt that the talks they had with colleagues were a type of psychological therapy. Some even testified that they had managed to grow based on the complex process they had undergone, as a type of staff unification process, as well as skill improvement. The nurses felt comfortable sharing their complex experiences with their co-nurses, as they were undergoing similar difficult experiences. One nurse claimed:

“Look, the people I work with understand me, the staff. Sometimes I also talk to family and friends, and they understand less. And even if they do understand, it’s not like the way the staff understands me. It’s different.”

(ICCU nurse, 27 years old, 5 months experience.)

Nurses sometimes feel that sharing and communicating with the staff regarding caring for COVID-19 patients is just like therapy, sitting on the psychologist’s couch. One nurse said:

“I mostly share with the staff. I have an amazing staff; we are each other’s psychologists. We deal with this thing together, as a group. We had some sort of powerful consolidation among the staff, and that’s really what made the work we did together easier, that’s what helped.”

(ICCU nurse, 26 years old, 12 months experience.)

Moreover, some nurses discovered positive points in this complex global issue, if that is even possible, such as consolidation and growth from very difficult, low places and the improvement of staff clinical skills.

“I think that the fact that we are experiencing this together, as a team, makes it much easier. It has helped us consolidate as a team, I think we discovered amazing abilities in each one of us, and our teamwork is also amazing. I always share with the staff because when we are on shift, we have the same experiences, so we have the same feelings, more or less.”

(ICCU nurse, 30 years old, 7 months experience.)

Another ICCU nurse demonstrated that:

“I think that the fact that we are experiencing this challenge in communication together allowed us, as a team, to look for other alternative ways for communication among us. Thus it helped us to be united as a team.”

(ICCU nurse, 26 years old, 9 months experience.)

The staff communicated and shared not only with the nursing staff but also with people close to them outside of work, mostly their spouses.

### 3.6. Sharing Feelings with People Close to Them Is a Source of Support

The staff felt that the people who understood them best were the people closest to them, particularly their spouses. Despite sharing with several people from the small and large family circles, some of the nurses still felt that what they had been undergoing was unbearable. One ICCU nurse claimed:

“Of course, I shared with my husband throughout. That’s obvious. He is my solid rock.”

(ICCU nurse, 31 years old, 7 months experience.)

Another nurse said:

“I talked a lot about it with my husband. He was the person who always supported me.”

(ICCU nurse, 37 years old, 5 months experience.)

The staff shared not only with the spouse but also with other household members and people from their wider social circle, such as friends, who lent them an ear. One nurse claimed:

“I shared with my husband and my friends, mostly my husband. He was always there. By the way, I shared with the children as well. They were always asking.”

(ICCU nurse, 31 years old, 10 months experience.)

“I mostly share with my household. My husband relates to my experiences. I talk about it a lot at home, that’s part of the things that helped me deal with these things, I always had someone to share with.”

(ICCU nurse, 31 years old, 8 months experience.)

The nursing staff felt comfortable sharing with their close circle the frustration they felt when working in the ICCU.

“I also talked to the extended family, I told them how difficult it was. In these situations, why people don’t keep safe. I thought about them when I went home, yes.”

(ICCU nurse, 27 years old, 10 months experience.)

Even though the staff felt comfortable sharing with family and colleagues, it seems that there are still complicated feelings and thoughts they preferred to leave unmentioned. An ICCU nurse said:

“I talk to my wife, she is also a nurse, and she is in charge of ventilated patients. No, the feelings, I am extremely charged, I’m finding the situation very difficult, what I went through in the ICCU during the pandemic.”

(ICCU male-nurse, 30 years old, 7 months experience.)

## 4. Discussion

This study sought to explore the experience of nurses’ communication challenges and strengths as they cared for COVID-19 intensive care patients during the two first pandemic waves. Analysis of the interviews yielded four sub-themes. The first two themes describe the challenges of communication between caregivers and COVID-19 intensive care patients, specifically difficulty communicating with patients due to the physical protection used and their medical condition. However, the other two themes describe the strength of communication as a support mechanism through sharing feelings with other caregivers and family. The four themes are discussed below.

The first major theme identified in our study was the challenge in communicating with patients due to personal protective equipment (PPE). Healthcare providers were required to enter the ICCU room wearing personal protective equipment (PPE), including but not limited to masks, face shields, gowns, and gloves, all essential for their safety [36,37]. Similarly, other researchers found that in face-to-face dialogues, physical obstacles, including a separation between the individuals and the wearing of face masks, impose new types of barriers regarding both verbal and nonverbal communication [21]. The face mask and face shield worn by caregivers of COVID-19 patients reduce the volume and clarity of spoken language. Additionally, these patients may be relying on lipreading to successfully understand others, a strategy that becomes lost when a face mask is used [21]. Visors and facemasks make it hard to hear gentle voice tones or read facial expressions, which are essential tools in empathetic communication [38]. For both patients and caregivers, these constraints resulted in a significant emotional toll. Patients’ distress and fear of the virus intensified when unable to identify caregivers covered head-to-toe in protective equipment [37]. Moreover, providing care while dressed in burdensome PPE changed healthcare delivery, reducing communication with patients and jeopardizing it due to the masks and shields [39].

The second major theme identified in our study was the challenge in communicating with patients due to their medical conditions. Similar to our findings, another study claimed that regular contact was being challenged by a high volume of patients receiving care and infection prevention concerns. It was very hard (both psychologically and for clear communication) for patients, relatives, and staff to have no eye-to-eye communication [40]. Patients’ worry and fear of the virus increased when unable to recognize caregivers covered head-to-toe in protective equipment [37]. The caregivers had been exhausted under layers of protective equipment, overworked due to increased demand in critical care and infected patients with unusual medical conditions, and anxious about how they were caring for their patients in this unusual setting [41]. The COVID-19 pandemic calls for bedside education, enhancements in compassion, and enhanced patient communication regarding caregivers [37]. During this time, professional resources have challenged ICCUs to continue to provide high-quality direct clinical treatment [40].

These challenges might be addressed by employing models of communication. The TAGEET communication model [40] may be of particular relevance in overcoming some of the challenges participants identified, as it encourages an awareness-based approach to communication and facilitates psychological self-care [42].

However, the nurses demonstrated not just challenges but also strengths. The third major theme identified in our study was sharing feelings with the staff as a source of support. The ICCU nurses felt that those who understood them most were the people who had similar experiences—their fellow nurses. Another study reported similar results, demonstrating that leadership communication in departments decreased moral distress among COVID-19 caregivers. An efficient manager communicating with employees during a crisis may lessen moral distress [18]. An additional study reinforced this insight, arguing that leadership communication is the most important tool, with optimal communication including openness, correctness, timeliness, understanding, and satisfaction [43]. Indeed, leaders should develop standard unit policies for crisis management. Implementing this may encourage a relationship before a crisis between nurses and managers, increasing the possibility for more open and efficient communication when a crisis occurs [18].

The fourth major theme identified in our study was sharing feelings with people close to them as a source of support. The ICCU nurses felt that those who helped them cope with the stressful time the most were people from their very close family circle and, particularly, their spouses. Similarly, another study found that the most common response of ICCU nurses to the question of what helped them during this time was support from co-workers and family/friends [44]. Furthermore, a study conducted among Australian volunteer firefighters suggested that support from family and friends was a crucial source for coping with the needs related to work and may safeguard them from burnout while helping them to stay connected to work volunteering [45]. It is worth mentioning that in the last few decades, there has been increased research studying the impact of home/work role conflict on health care providers [46] as they have great responsibility for patients’ lives. A study conducted with hospital employees found that whenever an individual perceived a proper balance between his work and personal life, a significant promotion occurred in different aspects of health [47]. Therefore despite knowing that sharing feelings with people could serve as a source of support, it is important to maintain a balance between work and personal life since this might be connected to nurses’ quality of life.

### 4.1. Strengths and Limitations and Future Studies

The study’s strengths are the in-depth new insights into ICCU nurses’ experience regarding their communication challenges and strengths as they cared for COVID-19 intensive care patients during the two first pandemic waves. Hence, the study results might have significant implications for other health professionals who care for COVID-19 intensive care patients.

There are several limitations to this study: one limitation is the small sample and the data collected from one area, from the center of the country, so there is no generalization ability to worldwide areas. Therefore, there is a need to examine in larger samples and internationally. Another limitation is the use of a single research tool, interviews. Therefore, future studies should concentrate on several tools, such as surveys, which can be useful for examining more vast insights.

### 4.2. Implications to Practice and Policy

There is a necessity to create an advanced plan, considering it provides vital practice and skills for nurses to be able to cope with such communication challenges and situations that posed sudden demands on patient care and facilitated nurses’ resilience [44]. Moreover, policymakers and governments need to create support systems, such as professional psychological counseling, continually, which may prevent burnout and allow them to continue the vital provision of patient care during the COVID-19 pandemic [48]. Additionally, they need to provide adequate information to remove ambiguity and provide educational support emphasizing humanism [41], especially related to crisis times.

## 5. Conclusions

The main research aim was to explore the lived experience of nurses’ communication challenges and strengths as they cared for COVID-19 intensive care patients during the two first pandemic waves. The nurses’ experiences described in this study illustrate their perceptions regarding the communications challenges and strengths while caring for patients with COVID-19.

The first two themes expressed the difficulties they experienced in communicating with patients due to the protective gear used and the patients’ medical condition. Thus, it is recommended to educate caregivers in this situation to speak slowly, use simple language, and purposely pause to verify comprehension. Since, under these undefined and very stressful circumstances, there is a continual need to keep efficient communication with the patients, the caregiver’s approach should be empathetic to relieve patients’ stress by acknowledging that they are cared for.

The other two themes expressed communication’s strengths as a support mechanism through sharing feelings with other caregivers and family. Caregivers need to talk about their experience working and communicating with COVID-19 patients. Therefore, it is recommended hospitals focus on providing more psychological support to nurses and adopt better training methods in the usage of coping strategies. It needed to create an optimistic environment for caregivers’ bonding, thus guaranteeing nurses’ mental safety and enabling them to win the battle against this epidemic.

## Figures and Tables

**Table 1:** Participants’ Background Information.

Variables		N = 22	
		Frequencies	Percentages
Gender	Male	5	27
	Female	17	73
Marital Status	Single	3	13
	Married	17	78
	Divorced	2	9
Education	B.A.	11	50
	M.A.	11	50
Role in the ICCU	Regular Nurse	20	91
	Clinical Instructor	1	4.5
	Nurse in Charge	1	4.5

**Table 2:** Interview questions.

1.	What is the communication experience of ICCU nurses in the COVID-19 battle?
2.	Describe the communication process during your shift in the ward when working with a COVID-19 patient?
3.	What are the main challenges or strengths you have experienced while working with a COVID-19 patient?
4.	What are your feelings regarding patient-caregiver communication when working with COVID-19 patients?
5.	Describe one case in which you had negative feelings and one case in which you had positive feelings regarding this communication process.
6.	Do communication thoughts (good or bad) related to your patients and their families follow you after the end of a shift?
7.	How does your environment react and communicate with you when they know that you work with COVID-19 patients?
8.	Were there any complex communication situations you required to manage throughout the pandemic?
9.	Did the pandemic create changes in your life? If so, how did you deal with them?
10.	Do you share the feelings you experienced as ICCU nurses in the COVID-19 ward?

## Data Availability

Not applicable.
